# Beta-lactam dosing in critically ill patients with septic shock and continuous renal replacement therapy

**DOI:** 10.1186/cc13938

**Published:** 2014-06-23

**Authors:** Marta Ulldemolins, Sergi Vaquer, Mireia Llauradó-Serra, Caridad Pontes, Gonzalo Calvo, Dolors Soy, Ignacio Martín-Loeches

**Affiliations:** 1 Fundació Clínic per la Recerca Biomèdica, Villarroel 170, 08036 Barcelona, Spain; 2 Critical Care Department, Corporación Sanitaria Universitaria Parc Tauli, Sabadell University Hospital, Parc Taulí 1, 08208 Sabadell, Barcelona, Spain; 3 School of Medicine, Universitat de Barcelona, Casanova 143, 08036 Barcelona, Spain; 4 Critical Care Department, Joan XXIII University Hospital, Institut d’Investigació Sanitària Pere Virgili, Doctor Mallafre Guasch 4, 43007 Tarragona, Spain; 5 Nursing Department, Universitat Rovira I Virgili, Avinguda Catalunya 35, 43002 Tarragona, Spain; 6 Clinical Pharmacology Department, Corporación Sanitaria Universitaria Parc Tauli, Sabadell University Hospital, Parc Taulí 1, 08208 Sabadell, Barcelona, Spain; 7 Pharmacology, Therapeutics and Toxicology Department, Campus Bellaterra, Universitat Autònoma de Barcelona, Sabadell, Spain; 8 Department of Clinical Pharmacology, Hospital Clínic de Barcelona, Villarroel 170, 08036 Barcelona, Spain; 9 Institut d’Investigacions Biomèdiques August Pi i Sunyer, Rosselló 149-153, 08036 Barcelona, Spain; 10 Pharmacy Department, Hospital Clinic de Barcelona, Villarroel 170, 08036 Barcelona, Spain; 11 Centro de Investigación Biomédica En Red de Enfermedades Respiratorias, Madrid, Spain

## Abstract

Although early and appropriate antibiotic therapy remains the most important intervention for successful treatment of septic shock, data guiding optimization of beta-lactam prescription in critically ill patients prescribed with continuous renal replacement therapy (CRRT) are still limited. Being small hydrophilic molecules, beta-lactams are likely to be cleared by CRRT to a significant extent. As a result, additional variability may be introduced to the *per se* variable antibiotic concentrations in critically ill patients. This article aims to describe the current clinical scenario for beta-lactam dosing in critically ill patients with septic shock and CRRT, to highlight the sources of variability among the different studies that reduce extrapolation to clinical practice, and to identify the opportunities for future research and improvement in this field. Three frequently prescribed beta-lactams (meropenem, piperacillin and ceftriaxone) were chosen for review. Our findings showed that present dosing recommendations are based on studies with drawbacks limiting their applicability in the clinical setting. In general, current antibiotic dosing regimens for CRRT follow a one-size-fits-all fashion despite emerging clinical data suggesting that drug clearance is partially dependent on CRRT modality and intensity. Moreover, some studies pool data from heterogeneous populations with CRRT that may exhibit different pharmacokinetics (for example, admission diagnoses different to septic shock, such as trauma), which also limit their extrapolation to critically ill patients with septic shock. Finally, there is still no consensus regarding the %T_>MIC_ (percentage of dosing interval when concentration of the antibiotic is above the minimum inhibitory concentration of the pathogen) value that should be chosen as the pharmacodynamic target for antibiotic therapy in patients with septic shock and CRRT. For empirically optimized dosing, during the first day a loading dose is required to compensate the increased volume of distribution, regardless of impaired organ function. An additional loading dose may be required when CRRT is initiated due to steady-state equilibrium breakage driven by clearance variation. From day 2, dosing must be adjusted to CRRT settings and residual renal function. Therapeutic drug monitoring of beta-lactams may be regarded as a useful tool to daily individualize dosing and to ensure optimal antibiotic exposure.

## Introduction

Optimal antibiotic dosing in the ICU is still a controversial issue that clinicians face daily. Despite compelling evidence supporting early and appropriate antibiotic therapy as one of the most effective interventions for improving patient outcome [1], antibiotic selection and dosing is often challenging in critically ill patients because of disease complexity, resulting physiological alterations, and reduced antibiotic susceptibilities of nosocomial pathogens. Besides, selecting an antimicrobial to which the causal agent is susceptible is not sufficient to achieve the best clinical outcomes, and factors such as adequate tissue penetration and achievement of a pharmacodynamic target associated with therapeutic success according to the antibiotic class are crucial for improving infection cure and patient morbi-mortality [2-4].

Beta-lactam antibiotics are time-dependent antibiotics, meaning that they exhibit optimal killing activity when plasma concentrations are maintained above the minimum inhibitory concentration of the bacteria during a percentage of the dosing interval (%T_>MIC_). Beta-lactams are also the most prescribed antibiotics in the ICU [5]. Significant and unpredictable pharmacokinetic variability of this pharmacological group has been well documented in critically ill patients: the volume of distribution (Vd) and the clearance (CL) of beta-lactams have been found to vary significantly depending on patient severity, proteinemia and organ failure, among other factors [3,6]. Acute kidney injury and the requirement of continuous renal replacement therapy (CRRT) add further variability to beta-lactam CL. However, available clinical evidence supporting beta-lactam dosing in critically ill patients with septic shock and CRRT is not yet optimal, since recommendations are mainly elucidated from healthy volunteers’ data and from clinical studies with important patient variability and limited sample sizes.

The aims of this article are to describe the current clinical scenario of beta-lactam dosing in critically ill patients with septic shock and CRRT, to highlight the sources of variability among the different studies that reduce extrapolation to clinical practice, and to identify the opportunities for future research and improvement in this field. For this purpose, two of the most frequently prescribed beta-lactams for nosocomial infections (meropenem and piperacillin) and a highly protein-bound antibiotic usually prescribed for community-acquired infections (ceftriaxone) were chosen for a thorough review. A systematic review of all available data on beta-lactam antibiotic pharmacokinetics in critically ill patients with CRRT was beyond the scope of this article, as this has been done elsewhere [7-9].

## Search strategy and selection criteria

Data for this review were identified by systematic searches of PubMed (1966 to November 2013), as well as references cited by relevant articles. Search terms included were ‘meropenem’ or ‘piperacillin’ or ‘ceftriaxone’, ‘critically ill patient’ or ‘intensive care unit’ or ‘critical illness’, ‘continuous veno-venous hemodiafiltration’ or ‘continuous veno-venous hemodialysis’ or ‘continuous veno-venous hemofiltration’ or ‘continuous renal replacement therapy’, and ‘pharmacokinetics’ or ‘pharmacodynamics’. Relevant articles written in English, Spanish and Catalan where considered for this review. Those articles describing the pharmacokinetics of meropenem, piperacillin/tazobactam and ceftriaxone in adult critically ill patients receiving CRRT were included.

## Effect of septic shock and CRRT in antibiotic dosing optimization

Classically, the *in vitro* susceptibility of the causal pathogen has been the cornerstone of antibiotic prescription. However, selection according to susceptibility is only a component of the optimal antibiotic therapy, and many other factors must also be considered. In terms of posology, it is paramount to design dosing strategies that maximize the likelihood of attaining the pharmacodynamic target associated with therapy success in the biophase. This is complex in the critically ill patient with septic shock and CRRT since it is well known that critical sickness and clinical interventions can drive to physiological changes likely to alter drug pharmacokinetics [3] and therefore likely to compromise the attainment of these pharmacodynamic targets.

There are two important time periods that must be considered for antibiotic dosing. The first period corresponds to the first day of therapy, where the main determinant for dosing must be the Vd since this determines the early attainment of antibiotic concentrations within the therapeutic range. In critically ill patients with sepsis, increased Vd must be expected for hydrophilic antibiotics such as beta-lactams (see Tables 1, 2, 3, 4, 5 and 6), aminoglycosides and glycopeptides [10-38]. This increase may be due to the presence of bacterial endotoxins in the bloodstream, which has a cascade effect on the production of endogenous molecules that act on the vascular endothelium, leading to vasodilation and transcapillary leakage of fluid and proteins into the extracellular space, where these antibiotics distribute. When the Vd is abnormally increased, distribution of hydrophilic antibiotics such as beta-lactams becomes more extensive for trying to compensate this larger space, with greater movement of the drug molecules from the central compartment (bloodstream) to the peripheral compartments (mainly extravascular fluid). The amount of the drug in plasma consequently decreases, and therefore the plasma concentration decreases. Consequently, given a particular minimum inhibitory concentration, shorter %T_>MIC_ values can be expected, which in turn may compromise beta-lactams’ pharmacodynamic target attainment [39]. Critically ill patients may therefore require front-loaded doses of beta-lactam antibiotics during the first 24 to 48 hours, regardless of organ function, in order to compensate the increased Vd and to reach concentrations within the therapeutic range on the first day of therapy [39].

**Table 1:** Available data on meropenem pharmacokinetics in continuous renal replacement therapy

**Study**	***n***	**Population and score**^**a**^	**Site of infection**	**Pathogen (MIC)**	**Antibiotic**	**Type of filter**	**Type of CRRT**	**RRT dose**^**a**^
Spanish product information	N/R	Healthy volunteers	N/A	N/A	Meropenem 2 g	N/A	N/A	N/A
Ververs and colleagues [16]	5	Critically ill patients with septic shock and AKI. No severity score reported	Several	Target: 100 % T_>MIC90_ of sensitive trains (*Serratia* sp. 0.06 mg/l and *Pseudomonas aeruginosa* 2 mg/l)	Meropenem 500 mg every 12 hours	PAN 06 polyacrylonitrile fiber membrane	CVVHF	Q_R_: 1.60 l/hour
Bilgrami and colleagues [15]	10	Critically ill patients with septic shock and AKI. APACHE II score 25 (22 to 28)	Several	Target: 100 % T_>MIC90_ of *Burkholderia pseudomallei* (MIC 4 mg/l)	Meropenem 1 g every 8 hours	AN 69 HF, 2.15 m^2^ polyacrylonitrile fiber membrane	CVVHF	Q_R_: 4.40 l/hour
Krueger and colleagues [24]	8	Critically ill patients with sepsis and MODS or cardiogenic shock and AKI. APACHE II score 29.90 ± 6.64	Several	Target: 40 % T_>MIC_ of susceptibility and intermediate-susceptibility breakpoint (4 and 8 mg/l, NCCLS)	Meropenem 500 mg every 12 hours	AN 69 HF, 0.9 m^2^ polyacrylonitrile fiber membrane	CVVHF	Q_R_: 1.60 l/hour
Thalhammer and colleagues [18]	9	Critically ill patients with sepsis and AKI. No severity score reported	Several	Target: 40 to 50 % T_>MIC90_ of *P. aeruginosa* susceptibility and intermediate-susceptibility breakpoint (4 and 8 mg/l, NCCLS)	Meropenem 1 g single dose	0.43 m^2^ polysulphone fiber membrane	CVVHF	Q_R_: 2.75 l/hour
Tegeder and colleagues [19]	9	Critically ill patients with septic shock and AKI. No severity score reported	Several (66.6 % abdominal)	Target: 100 % T_>MIC90_ of *P. aeruginosa* intermediate-susceptibility breakpoint (8 mg/l)	Meropenem 500 mg every 8 to 12 hours	AN 69 HF type of membrane N/R	CVVHF	Q_R_: 1 l/hour
Valtonen and colleagues [49]	6	Infected patients with AKI. No severity score reported	N/R	Target: 100 % T_>MIC90_ of *P. aeruginosa* and *Enterococcus faecalis* susceptibility breakpoint (4 and 8 mg/l, BSAC)	Meropenem 1 g single dose	AV 400S, 0.7 m^2^ polysulphone fiber membrane	CVVHDF	Q_D_: 1 l/hour, Q_R_: N/R
							CVVHDF	Q_D_: 2 l/hour, Q_R_: N/R
							CVVHF	Q_R_: N/R
Robatel and colleagues [20]	13	Critically ill patients with septic shock and AKI. No severity score reported	Several	Target: ≥75 % T_>MIC90_ of susceptibility breakpoint (4 mg/l)	Meropenem 0.5 to 1 g every 8 to 12 hours	AN 69 HF, 0.9 m^2^ polyacrylonitrile fiber membrane	CVVHDF	Q_D_: 0.60 to 1.50 l/hour, Q_R_: 0 to 1 l/hour
Langgartner and colleagues [21]	6	Critically ill patients with sepsis and AKI. No severity score reported	Several (50 % pneumonia)	Target: 100 % T_>MIC_*P. aeruginosa* intermediate-susceptibility breakpoint (MIC 8 mg/l)	Meropenem 1 g every 12 hours (bolus or CI)	AV 600S, 1.4 m^2^ polysulphone fiber membrane	CVVHDF	Total flow rate (Q_D_ + Q_R_): 2 l/hour
Seyler and colleagues [22]	17	Critically ill patients with severe sepsis/septic shock and AKI. No severity score reported	N/R	Target: 40 % T_>4xMIC_ of *P. aeruginosa* susceptibility breakpoint (≤2 mg/l, EUCAST) (8 mg/l)	Meropenem 1 g every 12 hours	AN 69 HF type of membrane N/R	CVVHDF / CVVHF	Q_D_: 1.61 ± 0.63, Q_R_: 1.54 ± 0.84 (for a 70 kg adult, weight not reported)
Giles and colleagues [23]	5	Critically ill patients with septic shock and AKI	N/R	Target: 100 % T_>MIC90_ of *P. aeruginosa* susceptibility breakpoint (4 mg/l)	Meropenem 1 g every 12 hours	AN 69 HF, 0.9 m^2^ polyacrylonitrile fiber membrane	CVVHF	Q_D_: 1.20 l/hour, Q_R_: 1.45 l/hour
	5	Critically ill patients with septic shock and AKI	N/R	Target: 100 % T_>MIC90_ of *P. aeruginosa* susceptibility breakpoint (4 mg/l)	Meropenem 1 g every 12 hours	AN 69 HF, 0.9 m^2^ polyacrylonitrile fiber membrane	CVVHDF	
Krueger and colleagues [17]	9	Critically ill patients with septic shock/cardiogenic shock and AKI. APACHE II 28.6 ± 9.1	Several (66.7 % pneumonia)	Target: 100 % T_>MIC_ of susceptibility and intermediate-susceptibility breakpoint (4 and 8 mg/l)	Meropenem 1 g every 12 hours	AN 69 HF, 0.9 m^2^ polyacrylonitrile fiber membrane	CVVHDF	Q_D_: 1.60 l/hour, Q_R_: variable
Isla and colleagues [26]	7	Critically ill patients with sepsis and CrCL <10 ml/minute. SOFA 13 ± 4.12	N/R	Target: 100 % T_>MIC90_ of *P. aeruginosa* and *Enterobacteriaceae* spp. susceptibility breakpoint (4 mg/l, NCCLS)	Meropenem 500 mg every 6 hours (5 cases), 500 mg every 8 hours (1 case), 1 g every 8 hours (1 case)	AN 69 HF 0.9 m^2^ polyacrylonitrile fiber/AV600S 1.4 m^2^ polysulphone fiber membrane	CVVHDF	Q_D_: 0.93 l/hour, Q_R_: 1.20 l/hour
	7	Critically ill patients with sepsis and CrCL 10 to 50 ml/minute. SOFA 12.3 ± 3.2	N/R	Target: 100 % T_>MIC90_ of *P. aeruginosa* and *Enterobacteriaceae* spp. susceptibility breakpoint (4 mg/l, NCCLS)	Meropenem 500 mg every 6 hours (6 cases), 1 g every 8 hours (1 case)	AN 69 HF 0.9 m^2^ polyacrylonitrile fiber/AV600S 1.4 m^2^ polysulphone fiber membrane	CVVHF (4 cases) / CVVHDF (3 cases)	Q_D_: 0.43 l/hour, Q_R_: 1.84 l/hour
	6	Critically ill patients (mostly trauma patients) with sepsis and CrCL >50 ml/minute. SOFA 14.0 ± 5.2	N/R	Target: 100 % T_>MIC90_ of *P. aeruginosa* and *Enterobacteriaceae* spp. susceptibility breakpoint (4 mg/l, NCCLS)	Meropenem 2 g every 8 hours (5 cases), 1 g every 6 hours (1 case)	AN 69 HF, 0.9 m^2^ polyacrylonitrile fiber membrane	CVVHF	Q_R_: 1.25 l/hour
Isla and colleagues [25]	13	Critically ill patients with sepsis and AKI. SOFA 11.9 ± 2.8	N/R	Target: 100 % T_>MIC90_ of *Enterobacteriaceae* spp., *P. aeruginosa* and *Staphylococcus aureus* susceptibility and intermediate-susceptibility breakpoints (4 and 8 mg/l respectively, NCCLS)	Meropenem 500 mg, 1 to 2 g every 6 to 8 hours	AN 69 HF, 0.9 m^2^ polyacrylonitrile fiber membrane or AV 600S, 1.4 m^2^ polysulphone fiber membrane	CVVHF / CVVHDF	Total flow rate (Q_D_ + Q_R_): 2.28 l/hour
Meyer and colleagues [27]	1	Critically ill patient with septic shock and AKI	Meningitis	Target: 100 % T_>MIC90_ of *Neisseria meningitidis* susceptibility breakpoint (0.016 mg/l)	Meropenem 1 g every 12 hours for three doses then 1 g every 8 hours	AN 69 HF, type of membrane N/R	CVVHDF	Q_D_: 0.75 l/hour, Q_R_: 1.25 l/hour

The table includes healthy volunteers’ data with comparative purpose. AKI, acute kidney injury; APACHE, Acute Physiology and Chronic Health Evaluation; BSAC, British Society for Antimicrobial Chemotherapy; CI, continuous infusion; CrCL, creatinine clearance; CRRT, continuous renal replacement therapy; CVVHDF, continuous venovenous hemodiafiltration; CVVHF, continuous venovenous hemofiltration; EUCAST, European Committee on Antimicrobial Susceptibility Testing; MIC, minimum inhibitory concentration; MODS, multiple organ dysfunction syndrome; N/A, not applicable; NCCLS, National Committee of Clinical Laboratory Standards; N/R, not reported; Q_D_, dialysis fluid flow rate; Q_R_, replacement fluid flow rate; RRT, renal replacement therapy; SOFA, Sequential Organ Failure Assessment; %T_>MIC_, percentage of dosing interval while concentration of the antibiotic is above the minimum inhibitory concentration of the pathogen. ^a^Data presented as mean ± standard deviation or median (interquartile range).

**Table 2:** Available data on meropenem pharmacokinetics in continuous renal replacement therapy

**Study**	**Sieving coefficient**^**a**^	**Type of pharmacokinetic analysis**	**Total CL (l/hour)**^**a**^	**Vd (l/kg)**^**a**^	**Residual diuresis (ml/24 hours)**^**a**^	**Clinical outcome**	**Authors’ dose recommendation**	**Study limitations**
Spanish product information	N/A	N/R	12.3	0.25	Normal renal function	N/A	N/A	N/A
Ververs and colleagues [16]	0.63 ± 0.252	Noncompartmental	4.57 ± 0.89	0.37 ± 0.15	Anuric (range 0 to 19 ml/24 hours)	20 % survival. 100 % target attainment	500 mg every 12 hours for sensible strains, shorter dosage interval for intermediate strains	No severity score reported, small sample size
Bilgrami and colleagues [15]	0.74 (0.71 to 0.77)	Noncompartmental	6 (5.2-6.2)	0.37 (0.32-0.46)	Oligoanuric	70 % survival. 100 % target attainment	1 g every 8 hours	High intensity used, not applicable to patients with standard CVVHF settings
Krueger and colleagues [24]	0.91 ± 0.1	Two-compartment modeling	4.98 ± 1.29	0.28 ± 0.07	<500	62.5 % survival. 100 % target attainment for MIC = 4 mg/l, 75 % target attainment for MIC = 8 mg/l	500 mg every 12 hours for susceptible bacteria	Heterogenic group with patients with cardiogenic shock
Thalhammer and colleagues [18]	N/R	Noncompartmental	8.62 ± 1.12	0.34 ± 0.03	Anuric	33.3 % survival. 100 % target attainment for MIC = 8 mg/l	1 g every 8 hours	First-dose pharmacokinetics, no severity score reported, no septic shock
Tegeder and colleagues [19]	1.17 ± 0.11	Noncompartmental	3.12 ± 0.50	0.18 ± 0.03 (for a 70 kg adult, weight not reported)	Five anuric, four with urine output <300 ml/24 hours	Survival N/R, 100 % target attainment	500 mg every 12 hours or 250 mg every 6 hours	No severity score reported
Valtonen and colleagues [49]	N/R	Noncompartmental	4.72 ± 2.69	N/R	111.8 ± 201.7	Survival N/R, 83.3 % target attainment	1 g every 12 hours	No report of Vd. First-dose pharmacokinetics. No septic shock, not applicable to critically ill patients
	N/R	Noncompartmental	5.71 ± 3.58	N/R	120.9 ± 204.7	Survival N/R, 83.3 % target attainment	1 g every 12 hours	No report of Vd. First-dose pharmacokinetics. No septic shock, not applicable to critically ill patients
	N/R	Noncompartmental	3.27 ± 2.30	N/R	120.9 ± 204.7	Survival N/R, 83.3 % target attainment	500 mg every 8 hours	No report of Vd. First-dose pharmacokinetics. No septic shock, not applicable to critically ill patients
Robatel and colleagues [20]	0.65 (39 % CV)	Four-compartment modeling	5.5 (38 % CV)	0.52	Anuric	46.7 % survival. Pharmacokinetic target attainment N/R	750 mg every 8 hours or 1.5 g every 12 hours	No severity score reported, no average total CRRT dose reported
Langgartner and colleagues [21]	0.97 (0.87 to 1.05), bolus 0.89 (0.79 to 0.93), CI	Noncompartmental	4.32 (3.93 to 4.96), bolus 4.40 (3.58 to 5.58), CI	0.43 (0.38 to 0.54)	N/R	66.7 % survival. 83.3 % target attainment in CI, 66.6 % target attainment in bolus	500 mg loading dose, 2 g every 24 hours CI	No severity score and residual renal function reported, no septic shock
Seyler and colleagues [22]	N/R	Noncompartmental	4.9 (2.1 to 14) (for a 70 kg adult, weight N/R)	0.45 (0.20 to 3.03)	N/R	Survival N/R, 81 % target attainment	1 g every 8 hours loading dose (first 48 hours), dose reduction thereafter	CVVHDF and CVVHF data analyzed altogether. No severity score and residual renal function reported
Giles and colleagues [23]	0.95 ± 0.03	Two-compartment modeling	3.63 ± 0.95	0.38 ± 0.12	N/R	60 % survival, 60 % target attainment	1 g every 12 hours	Small sample size. No residual renal function reported.
	0.91 ± 0.09	Two-compartment modeling	4.72 ± 1.69	0.31 ± 0.08	N/R	60 % survival, 60 % target attainment	1 g every 12 hours	Small sample size. No residual renal function reported.
Krueger and colleagues [17]	1.06	Two-compartment modeling	3.28 ± 1.02	0.26 ± 0.09	Anuric	66.7 % survival, 100 % target attainment	1 g every 12 hours	Heterogenic group with patients with cardiogenic shock. Q_D_ not reported
Isla and colleagues [26]	0.76 ± 0.10	Noncompartmental	9.0 ± 4.55	0.57 ± 0.29	N/R, mean CrCL = 1.1 ml/minute	Survival N/R, 85.7 % target attainment	500 mg every 6 hours	No septic shock. The study compares three groups with different CRRT modalities. No residual diuresis and CrCL estimation method reported
	0.85 ± 0.13	Noncompartmental	8.16 ± 3.43	0.37 ± 0.10	N/R, mean CrCL = 23.5 ml/minute	Survival N/R, 57.1 % target attainment	500 mg every 6 hours	No septic shock. CVVHDF and CVVHF data analyzed altogether. The study compares three groups with different CRRT modalities. No residual diuresis and CrCL estimation method reported
	N/R	Noncompartmental	63.90 ± 39.74	1.31 ± 0.9	N/R, mean CrCL = 95.9 ml/minute	Survival N/R, 16.7 % target attainment	Doses >2 g every 8 hours	No septic shock. Mainly trauma patients. The study compares three groups with different CRRT modalities. No residual diuresis and CrCL estimation method reported
Isla and colleagues [25]	0.72 (6.3 % CV)	Two-compartment modeling	8.04 (13 % CV)	0.50 (10 % CV)	N/R, mean CrCL = 22 ml/minute	Survival N/R, target attainment N/R	CI of 700 mg/24 hours (MIC = 4 mg/l) or 1,400 mg/24 hours (MIC = 8 mg/l) in CrCL <10 ml/minute, higher doses when >10 ml/minute	No septic shock. CVVHDF and CVVHF data analyzed altogether. Different filters used. No residual diuresis and CrCL estimation method reported
Meyer and colleagues [27]	1.02 ± 0.26	Noncompartmental	7.76	0.54	Anuric	Survived but with significant sequels. Pharmacodynamic target was attained	1 g every 12 hours	Case report with limited comparability to other studies

The table includes healthy volunteers’ data with comparative purpose. CI, continuous infusion; CL, clearance; CrCL, creatinine clearance; CRRT, continuous renal replacement therapy; CV, coefficient of variation; CVVHDF, continuous venovenous hemodiafiltration; CVVHF, continuous venovenous hemofiltration; MIC, minimum inhibitory concentration; N/A, not applicable; N/R, not reported; Q_D_, dialysis fluid flow rate; Vd, volume of distribution. ^a^Data presented as mean ± standard deviation or median (25 to 75 % range).

**Table 3:** Available data on piperacillin pharmacokinetics in continuous renal replacement therapy

**Study**	***n***	**Population and score**^**a**^	**Site of infection**	**Pathogen (MIC)**	**Antibiotic**	**Type of filter**	**Type of CRRT**	**RRT dose**^**a**^
Occhipinti and colleagues [28]	12	Healthy volunteers	N/A	N/A	Piperacillin 4.5 g every 8 hours	N/A	N/A	N/A
Arzuaga and colleagues [29]	4	Critically ill patients with sepsis and CrCL <10 ml/minute. SOFA 13.5 ± 3.1	Several	Target: 100 % T_>MIC_ for susceptibility and intermediate-susceptibility breakpoints (<32 mg/l and >64 mg/l)	Piperacillin/tazobactam 4.5 g every 6 to 8 hours	AN 69 HF, 0.9 m^2^ copolymer filter	CVVHF	Q_R_: 1.63 ± 0.47 l/hour
	5	Critically ill patients with sepsis and CrCL 10 to 50 ml/minute. SOFA 11 ± 2.1	Several (60 % peritonitis)	Target: 100 % T_>MIC_ for susceptibility and intermediate-susceptibility breakpoints (<32 and >64 mg/l)	Piperacillin/tazobactam 4.5 g every 6 to 8 hours	AN 69 HF, 0.9 m^2^ copolymer filter	CVVHF	Q_R_: 1.82 ± 0.26 l/hour
	5	Critically ill patients with sepsis and CrCL >50 ml/minute. SOFA 9 ± 1.4	Several (60 % VAP)	Target: 100 % T_>MIC_ for susceptibility and intermediate-susceptibility breakpoints (<32 and >64 mg/l)	Piperacillin/tazobactam 4.5 g every 6 to 8 hours	AN 69 HF, 0.9 m^2^ copolymer filter	CVVHF	Q_R_: 1.20 ± 0.45 l/hour
van der Werf and colleagues [30]	9	Critically ill patients with septic shock and MODS. APACHE II 30.1 ± 4.2	Several	Target: 100 % T_>MIC_ of the *in vitro* sensitivity of microbial isolates recovered from the infection site	Piperacillin/tazobactam 4.5 g every 8 hours	N/R	CVVHF	Q_R_: 1.55 ± 0.59 l/hour
Capellier and colleagues [31]	10	Critically ill patients with septic shock (seven cases) or cardiogenic shock (three cases) and AKI. SAPS II score 74 ± 6	N/R	N/R	Piperacillin 4 g every 8 hours (six cases first dose, four cases steady state)	0.5 m^2^ polysulphone filter	CVVHF	N/R
Asín-Prieto and colleagues [32]	Total: 16, N/R by degree of renal function	Critically ill patients with sepsis/polytrauma and different degrees of renal function (CrCL 1.3 to 110 ml/minute). SOFA 11 ± 3	N/R	Target: 100 % T_>MIC_ for the susceptibility breakpoint (16 mg/dl) (CLSI)	Piperacillin/tazobactam 4.5 g every 4, 6 and 8 hours (two, seven and seven cases, respectively)	AN 69 HF, 0.9 m^2^ copolymer filter	CVVHF	Q_R_: 1.54 ± 0.43 l/hour
Bauer and colleagues [33]	42	Critically ill patients with sepsis and AKI/end-stage renal disease. CCF score 7.9 ± 2.8	N/R	Target: 50 % T_>MIC_ for the susceptibility and intermediate-susceptibility breakpoint (16 and 64 mg/dl)	Piperacillin/tazobactam 2.25 to 3.375 g every 6, 8 and 12 hours	M60 to M100 HF, 0.6 to 0.9 m^2^ acrylonitrile filter or NxStage System One, 1.5 m^2^ polyethersulphone filter	CVVHD / CVVHDF	Q_T_: 2.4 (for mean weight of 95 kg)
Mueller and colleagues [34]	8	Critically ill patients with sepsis and AKI. No severity score reported	Pneumonia	Target: 50 % T_>MIC_ for the susceptibility and intermediate-susceptibility breakpoint (16 and 32 mg/dl)	Piperacillin/tazobactam 4.5 g every 8, 12 and 24 hours (three, four and one cases, respectively)	AN 69 HF, 0.6 m^2^ filter	CVVHD	Q_D_: 1.5 l/hour, Q_R_: 0.08 to 0.20 l/hour
Keller and colleagues [35]	12	Critically ill patients with sepsis and AKI. No severity score reported	Several	N/R	Piperacillin 4 g single dose (10 cases), 4 g every 8 hours (two cases)	AN 69 HF, 0.43 m^2^ copolymer filter	CAVHD	Q_D_: 1.22 ± 0.09 l/hour
Valtonen and colleagues [50]	6	Septic patients with AKI. No severity score reported	Several	Target: 100 % T_>MIC_*Pseudomonas* spp. and *Enterobacteriaceae* spp. susceptibility breakpoint (16 mg/dl, BSAC)	Piperacillin/tazobactam 4.5 g every 12 hours	AV 400S, 0.7 m^2^ polysulphone membrane	CVVHDF	Q_D_: 1 l/hour, Q_R:_ N/R
							CVVHDF	Q_D_: 2 l/hour, Q_R:_ N/R
							CVVHF	Q_R:_ N/R
Seyler and colleagues [22]	16	Critically ill patients with severe sepsis/septic shock and AKI. No severity score reported	N/R	Target: 50 % T_>4xMIC_*Pseudomonas aeruginosa* susceptibility breakpoint (≤16 mg/l, EUCAST) (64 mg/l)	Piperacillin/tazobactam 4.5 g every 6 hours	AN 69 HF, type of membrane N/R	CVVHDF/CVVHF	Q_D_: 0.023 ± 0.009 l/kg/hour (1.61 l/hour for a 70 kg adult, weight N/R), Q_R_: 0.022 ± 0.012 l/kg/hour (1.54 l/hour for a 70 kg adult, weight N/R)
Varghese and colleagues [38]	10	Critically ill patients with severe sepsis/septic shock and AKI. APACHE II 33 (31 to 36), SOFA 12 (10 to 15)	N/R	Target: 50 % T_>MIC_ for clinically relevant MIC (2, 4, 8, 16, 32 and 64 mg/l) in plasma and subcutaneous tissue	Piperacillin/tazobactam 4.5 g every 8 hours	AN 69 HF, 1.05 m^2^ polyacrylonitrile filter	CVVHDF	Q_D_: 1 to 1.5 l/hour, Q_R:_ 1.5 to 2 l/hour, Q_T_: 3.0 to 3.9 l/hour

The table includes healthy volunteers’ data with comparative purpose. AKI, acute kidney injury; APACHE, Acute Physiology and Chronic Health Evaluation; BSAC, British Society for Antimicrobial Chemotherapy; CAVHD, continuous arteriovenous hemodialysis; CCF, Cleveland Clinic Foundation; CI, continuous infusion; CLSI: Clinical and Laboratory Standards Institute; CrCL, creatinine clearance; CRRT, continuous renal replacement therapy; CVVHD, continuous venovenous hemodialysis; CVVHDF, continuous venovenous hemodiafiltration; CVVHF, continuous venovenous hemofiltration; EUCAST, European Committee on Antimicrobial Susceptibility Testing; MIC, minimum inhibitory concentration; MODS, multiple organ dysfunction syndrome; N/A, not applicable; N/R, not reported; Q_D_, dialysis fluid flow rate; Q_R_, replacement fluid flow rate; Q_T_, total flow rate; RRT, renal replacement therapy; SAPS, Simplified Acute Physiology Score; SOFA, Sequential Organ Failure Assessment; %T_>MIC_, percentage of dosing interval while concentration of the antibiotic is above the minimum inhibitory concentration of the pathogen; VAP, ventilator-associated pneumonia. ^a^Data presented as mean ± standard deviation or median (interquartile range).

**Table 4:** Available data on piperacillin pharmacokinetics in continuous renal replacement therapy

**Study**	**Sieving coefficient**^**a**^	**Type of pharmacokinetic analysis**	**Total CL (l/hour)**^**a**^	**Vd (L/kg)**^**a**^	**Residual diuresis (ml/24 hours)**^**a**^	**Clinical outcome**	**Authors dose recommendation**	**Study limitations**
Occhipinti and colleagues [28]	N/A	Noncompartmental	10.90 ± 1.17 l/hour/1.73 m^2^	0.15 ± 0.02	N/A	N/A	N/A	N/A
Arzuaga and colleagues [29]	0.42 ± 0.25	Noncompartmental	3.00 ± 3.22	0.28 ± 0.16	N/R, CrCL <10 ml/minute	Survival N/R, 100 % target attainment	Dose reduction	Small sample size, no residual diuresis and CrCL estimation method reported
	0.38 ± 0.37	Noncompartmental	5.44 ± 1.80	0.36 ± 0.27	N/R, CrCL 10 to 50 ml/minute	Survival N/R, 100 % target attainment for MIC < 32 mg/l, 50 % target attainment for MIC > 64 mg/l	Dose reduction	Small sample size, no residual diuresis and CrCL estimation method reported
	0.23 ± 0.07	Noncompartmental	15.91 ± 9.13	0.56 ± 0.25	N/R, CrCL >50 ml/minute	Survival N/R, 55.5 % target attainment for MIC < 32 mg/l, 16.6 % target attainment for MIC > 64 mg/l	4.5 g every 4 hours	Small sample size, no residual diuresis and CrCL estimation method reported
van der Werf and colleagues [30]	N/R	Two compartments	2.52 ± 1.38	0.30 ± 0.21	Anuric	77.8 % survival, 100 % target attainment	Dose as for patients with slightly impaired renal function	No report of sieving, no report of MIC (classified as S/R)
Capellier and colleagues [31]	N/R	Noncompartmental	First dose: 4.75 ± 1.42, steady state: 1.49 ± 0.79	First dose: 0.48 ± 0.24, steady state: 0.14 ± 0.07	Mainly anuric, three with residual diuresis between 220 and 400 ml/24 hours	N/R	4.5 g every 12 hours	No CRRT dose, MIC target and outcome reported, some patients with cardiogenic shock
Asín-Prieto and colleagues [32]	0.37 ± 0.25	Two compartments	7.32 (4.21 to 10.86) (bootstrap)	0.59 (0.38 to 0.82) (bootstrap)	Different degrees of renal function, residual diuresis N/R, CrCL 43 ± 34 ml/minute	Survival N/R, target attainment (MIC = 16 mg/l) after simulations: when CrCL >100 ml/minute, 60 % target attainment with high doses (4 g every 4 hours); when CrCL = 50 ml/minute, 93 % target attainment with 4 g every 4 hours, 62 % PTA with 4 g every 6 hours; when CrCL = 10 ml/minute, 96 % target attainment with 4 g every 8 hours	After simulations: when CrCL = 100 ml/minute, CI 16 g every 24 hours; when CrCL = 50 ml/minute, CI 12 g every 24 hours	No report of number of patients by renal function group, no report of residual diuresis, CrCL estimated using Cockroft–Gault method (not validated for critically ill patients)
Bauer and colleagues [33]	N/R	One compartment	3.87 l/hour (IQR: 3.56)	0.38 l/kg (IQR: 0.20)	Oligoanuric (median 38 ml/24 hours, IQR: 157 ml)	50 % survival, 100 % target attainment for MIC = 16 mg/l (total and unbound piperacillin), 83 % target attainment for MIC = 64 mg/l (total piperacillin), and 77 % target attainment (unbound)	>9 g piperacillin/day	Sparse sampling, CVVHDF and CVVHD data analyzed altogether
Mueller and colleagues [34]	0.84 ± 0.21	Noncompartmental	2.82 (1.56 to 13.2)	0.31 ± 0.07	Anuric	Survival N/R, simulations show 87.5 % target attainment with 4.5 g every 12 hours/2.25 g every 8 hours	4.5 g every 12 hours or 2.25 g every 8 hours	No severity score and outcomes reported, no septic shock
Keller and colleagues [35]	0.71 ± 0.21	One compartment	2.83 ± 1.34	0.37 ± 0.05 (for a 70 kg adult, weight N/R)	Anuric	16.7 % survival.	150 % of dose for anuric patients	First-dose kinetics, no severity score, MIC target and outcomes reported
Valtonen and colleagues [50]	N/R	Noncompartmental	5.06 ± 1.68	N/R	133 ± 199	Survival N/R, 33.3 % target attainment	4.5 g every 8 hours	No severity score and Vd reported. No septic shock, not applicable to critically ill patients
	N/R	Noncompartmental	5.48 ± 2.11	N/R	151 ± 224	Survival N/R, 33.3 % target attainment	4.5 g every 8 hours	No severity score and Vd reported. No septic shock, not applicable to critically ill patients
	N/R	Noncompartmental	3.89 ± 1.23	N/R	109 ± 182	Survival N/R, 33.3 % target attainment	4.5 g every 8 hours	No severity score and Vd reported. No septic shock, not applicable to critically ill patients
Seyler and colleagues [22]	N/R	Noncompartmental	4.9 (0.14 to 26.6) (for a 70 kg adult, weight N/R)	0.44 (0.22 to 1.72)	N/R	Survival N/R, 71 % target attainment	4.5 g every 6 hours loading dose (first 48 hours), dose reduction thereafter	CVVHDF and CVVHF data analyzed altogether. No severity score, weight and residual renal function reported
Varghese and colleagues [38]	0.67 (0.53 to 0.78)	Noncompartmental	5.1 (4.2 to 6.2)	0.42 (0.29 to 0.49)	Five anuric, five oliguric (<0.5 ml/kg/hour for ≥6 hours)	Survival N/R, 100 % target attainment for MIC ≤32 mg/l	4.5 g every 8 hours for susceptible microorganisms (MIC ≤32 mg/l)	No site of infection and survival reported

The table includes healthy volunteers’ data with comparative purpose. CI, continuous infusion; CL, clearance; CrCL, creatinine clearance; CRRT, continuous renal replacement therapy; CVVHD, continuous venovenous hemodialysis; CVVHDF, continuous venovenous hemodiafiltration; CVVHF, continuous venovenous hemofiltration; IQR, interquartile range; MIC, minimum inhibitory concentration; N/A, not applicable; N/R, not reported; PTA, probability of target attainment; S/R, sensitive/resistant; Vd, volume of distribution. ^a^Data presented as mean ± standard deviation or median (25 to 75 % range).

**Table 5:** Available data on ceftriaxone pharmacokinetics in hemofiltration

**Study**	***n***	**Population and score**^**a**^	**Site of infection**	**Pathogen (MIC)**	**Antibiotic**	**Type of filter**	**Type of CRRT**	**RRT dose**^**a**^
Spanish product information	N/R	Healthy volunteers	N/A	N/A	Ceftriaxone 1 g	N/A	N/A	N/A
Garot and colleagues [36]	54	Critically ill patients with sepsis, severe sepsis or septic shock with various degrees of renal function, 12 with CVVHF. SAPS II 50 (9 to 87)	Several (61 % pneumonia)	100 % T_>MIC_ for MIC values ranging from 0.016 mg/dl (*Streptococcus pneumoniae*) to 8 mg/dl (*Staphylococcus aureus*)	Ceftriaxone 2 g every 24 hours (41 cases), 1 g every 24 hours (one case), 2 g every 12 hours (one case) and 2 g every 8 hours (one case)	N/R	CVVHF	N/R
Kroh and colleagues [37]	6	Critically ill patients with sepsis and AKI	Several	N/R	Ceftriaxone 2 g every 24 hours	Polyamide filter	CVVHF	Q_R_: 1.2 to 1.8 l/hour

The table includes healthy volunteers’ data with comparative purpose. AKI, acute kidney injury; CRRT, continuous renal replacement therapy; CVVHF, continuous venovenous hemofiltration; MIC, minimum inhibitory concentration; N/A, not applicable; N/R, not reported; Q_R_, replacement fluid flow rate; RRT, renal replacement therapy; SAPS, Simplified Acute Physiology Score; %T_>MIC_, percentage of dosing interval while concentration of the antibiotic is above the minimum inhibitory concentration of the pathogen. ^a^Data presented as mean ± standard deviation or median (interquartile range).

**Table 6:** Available data on ceftriaxone pharmacokinetics in hemofiltration

	**Sieving coefficient**^**a**^	**Type of pharmacokinetic analysis**	**Total CL (l/hour)**^**a**^	**Vd (l/kg)**^**a**^	**Residual diuresis (ml/24 hours)**^**a**^	**Clinical outcome**	**Authors’ dose recommendation**	**Study limitations**
Spanish product information	N/A	N/R	0.6 to 1.2	0.10 to 0.17	N/A	N/A	N/A	N/A
Garot and colleagues [36]	N/R	Two compartments	0.97 (for low CrCL = 5.5 ml/minute)	0.26 (for a 70 kg adult, weight N/R)	N/R, CrCL range 5.5 to 214 ml/minute	100 % attainment of 100 % T_>MIC_	No dose adjustment	No report of severity scores, RRT settings, residual diuresis and CrCL estimation method, unbound concentration calculated using a formula, heterogenic population
Kroh and colleagues [37]	0.69 ± 0.39	Noncompartmental	2.36	0.42 ± 0.19	N/R, CrCL range 0 to 10 ml/minute	N/R	No dose adjustment	No residual diuresis and CrCL estimation method reported. No outcomes study performed, no septic shock, no albumin concentrations considered

The table includes healthy volunteers’ data with comparative purpose. CL, clearance; CrCL, creatinine clearance; N/A, not applicable; N/R, not reported; RRT, renal replacement therapy; %T_>MIC_, percentage of dosing interval while concentration of the antibiotic is above the minimum inhibitory concentration of the pathogen; Vd, volume of distribution. ^a^Data presented as mean ± standard deviation or median (25 to 75 % range).

The particular case of CRRT requirement poses another scenario where loading doses may be considered. At the time of CRRT initiation, antibiotic concentrations over time are in steady-state equilibrium (if the antibiotic was initiated before CRRT commencement), but one can hypothesize that the change in drug CL induced by CRRT initiation may lead to the breakage of this equilibrium and, consequently, to a decrease in drug concentrations. A new steady state will follow after seven half-lives since the introduction of the foreign source of drug CL. During this time period, however, concentrations may fall below the therapeutic range. At this point, an additional loading dose may help in the maintenance of therapeutic levels. This phenomenon of steady-state breakage follows the theoretical pharmacokinetics principles but there are no studies yet that describe it in critically ill patients and hence concrete loading dose recommendations cannot be provided. Certainly this is a very interesting area that deserves further research to be properly understood.

The second period starts from day 2. During this period, the estimated drug CL is the main determinant of dosing, with the objective of maintaining the equilibrium between input and output as the tissues should already hold therapeutic antibiotic concentrations. In this context, CRRT represents a particular challenge in terms of dosing, especially for hydrophilic antibiotics, as concentrations may vary depending on the degree of extraction, which in turn depends on the CRRT modality, on drug physicochemistry and, presumably, on CRRT intensity [7]. Moreover, residual renal function is usually variable, difficult to assess and rarely considered when dosing, despite its relevant contribution to antibiotic CL in patients undergoing CRRT that has been described for meropenem and piperacillin among others [26,29,32]. Finally, the patient’s condition evolves throughout the ICU stay so the influence of the previously mentioned factors may vary over time, making it difficult to generalize recommendations only based on CRRT modality and intensity. Dosing should ideally be titrated daily depending on the CRRT settings and the evolution of the patient’s renal function. With this aim, therapeutic drug monitoring (TDM) of trough levels might be a useful tool for refining dosing decisions during the maintenance phase of therapy, as it is routinely performed with aminoglycosides and glycopeptides. However, despite emerging data suggesting that beta-lactam TDM might improve the attainment of pharmacodynamic targets associated with therapeutic success [40], the impact of systematic TDM on clinical outcomes and resource use is still to be prospectively validated. Due to the variable pharmacokinetics of these drugs in critically ill patients with CRRT, TDM certainly deserves further investigation.

## Determinants of drug clearance by CRRT

Among the many options for renal replacement, CRRT is the most used in the critical care setting due to its advantages in hemodynamically unstable patients compared with intermittent techniques [41]. Drug clearance through CRRT is multifactorial and depends on both drug characteristics and CRRT modality and intensity. Continuous venovenous hemodialysis is based on the principle of diffusion of solutes across a semipermeable membrane driven by a concentration gradient, while continuous venovenous hemofiltration clearance is driven mainly by convection removal, where a positive hydrostatic pressure drives water and solutes across the filter membrane from the blood compartment to the filtrate compartment, from which it is drained. Continuous venovenous hemodiafiltration is the most efficient technique for solute removal, consisting of a combination between the two abovementioned techniques and resulting in the removal of hydrophilic solutes with simultaneous water elimination [7].

Regardless of the modality prescribed, a common determinant of drug clearance in CRRT is protein binding. Due to protein size and electrical charge, protein-bound molecules are unable to pass through the filter membranes and only unbound molecules will be available for elimination by CRRT. This is so critical that both sieving coefficients and saturation coefficients are usually simplified as the unbound drug fraction. However, antibiotic protein-binding alterations have been broadly observed in ICU patients [6] due to the altered plasmatic protein homeostasis associated with critical illness (the SAFE study reported that 40 to 50 % of the ICU patients had albumins <25 g/l) [42] and due to the presence of other highly protein-bound exogenous drugs and endogenous molecules (such as bilirubin) in plasma. This may consequently translate into alterations in the extent to which an antibiotic is cleared by CRRT. However, whereas the effect of hypoalbuminemia on antibiotic pharmacokinetics in critically ill patients with preserved renal function has been documented in previous studies [6], there are no available studies regarding its combined impact with CRRT.

Another factor likely to affect the extent to which drugs are cleared by CRRT is the CRRT intensity. The question of what is the optimal CRRT intensity has been a controversial issue since its first implantation. Several studies have evaluated the impact of using different CRRT intensities on mortality and recovery of renal function in critically ill patients, with different, usually debatable, results [43-48]. Due to this lack of definitive evidence, current clinical recommendations define the area of best practice for CRRT intensity as lying between 20 and 40 ml/kg/hour [41], the clinician being responsible for individualizing the appropriate CRRT intensity for each particular patient. However, the impact of different CRRT intensities on antibiotic dosing requirements has not yet been sufficiently evaluated.

Additional to the abovementioned points, more variability in drug CL by CRRT may be introduced by medical devices that may coexist with CRRT in patients with septic shock, such as polymyxin B fiber columns (to reduce endotoxin levels in sepsis) or extracorporeal membrane oxygenation. Other factors such as filter lifespan, filter anticoagulants such as citrate and drug recirculation may also have an effect on drug CL. However, their potential for antibiotic adsorption and removal has not yet been estimated.

## Main limitations of available pharmacokinetic studies

To discuss the current scenario of beta-lactam dosing in patients with septic shock and CRRT, we performed a thorough review of the existing clinical data for three of the most frequently used (and studied) beta-lactam antibiotics in the ICU. Tables 1, 2, 3, 4, 5 and 6 summarize the available evidence on meropenem, piperacillin/tazobactam and ceftriaxone pharmacokinetics in critically ill patients with CRRT [15-38,49,50].

Critical review of these studies has lead to identification of the following points that limit applicability of dose recommendations to critically ill patients with septic shock and CRRT.

### Patient population

The identified studies handle a highly heterogeneous patient population, which may jeopardize the generalizability of the results. For example, there are studies that pool together patients with septic shock and cardiogenic shock [17,31]. The physiopathology of these two types of shock, however, is very different: septic shock is caused by peripheral vasodilation, systemic inflammation and, consequently, increased Vd; while cardiogenic shock involves peripheral vasoconstriction, which should have no effect on the Vd.

Other studies include septic and polytrauma patients requiring CRRT [25,32]. Of note, one of these studies overcame the admission diagnosis-driven variability by developing a population pharmacokinetics model. The investigators found that admission diagnosis significantly influenced pharmacokinetic parameters: trauma patients exhibited higher Vd and CL than septic patients (d = 69.5 and 15.7 l in trauma patients and septic patients, respectively; CL = 54.15 and 8.04 l/hour in trauma patients and septic patients, respectively) [25]. Patients with sepsis/severe sepsis may also substantially differ from patients with septic shock: septic shock patients may exhibit higher Vd due to capillary leakage and aggressive fluid resuscitation as compared with critically ill patients without septic shock. In spite of this, some of the available studies include patients with sepsis/severe sepsis and acute kidney injury [21,33-35,37,49,50] but not those with septic shock.

Furthermore, a significant number of the articles do not report clinical severity scores for the studied population. In particular, increasing Acute Physiology and Chronic Health Evaluation II scores have been shown to correlate with increased Vd for hydrophilic antibiotics such as aminoglycosides [12]. However, variations in the Vd of meropenem and piperacillin have been reported in the literature (see Tables 1, 2, 3, 4 and 5). Similarly, CRRT may be prescribed in patients who still present a significant residual renal function. The influence of residual renal function on piperacillin pharmacokinetics in patients receiving continuous venovenous hemofiltration has been assessed by Arzuaga and colleagues, and significant differences in piperacillin CL have been reported; for example, total drug CL in patients with creatinine CL > 50 ml/minute was tripled as compared with patients with creatinine CL < 10 ml/minute [51]. These points suggest that the one-size-fits-all dosing recommendations based only on CRRT prescription may not apply to all different types of critically ill patients, as they are a highly heterogeneous population that may require different doses.

### Continuous renal replacement therapy modality and flow rate

Regarding CRRT modalities, there is discordance in the literature on whether a specific modality makes a difference or not in terms of dosing. While some studies support a difference in CL partially due to CRRT modality [49,50], some others suggest that there are no substantial variations between modalities [22]. Theoretically, convective and diffusive methods eliminate molecules from the bloodstream using different processes, and therefore the total drug CL should differ between CRRT modalities, as has been shown with piperacillin and meropenem [49,50], but a significant volume of dosing recommendations are still generic for CRRT.

Regarding CRRT intensity, emerging evidence suggests that the total flow rate affects the CL of hydrophilic drugs with low protein binding. For example, Beumier and colleagues developed a population pharmacokinetics model for vancomycin administered as a continuous infusion in critically ill patients with sepsis and septic shock, and found that inclusion of CRRT intensity as a covariate on CL significantly improved the model [52]. Similarly, a study by Bilgrami and colleagues specifically targeted patients with high-intensity CRRT (>4 l/hour) receiving meropenem and found that total drug CL was higher compared with previous studies with lower intensity CRRT, intensity being the main parameter that accounted for the differences in meropenem CL (*R*^2^ = 0.89) [15]. The high CRRT intensity was such a determinant of meropenem CL that the doses required for the coverage of less susceptible bacteria (minimum inhibitory concentration = 4 mg/l) were similar to those used in patients without renal failure (1,000 mg every 8 hours). These data suggest that different CRRT intensities may translate into different drug CL and therefore into different dose requirements. Importantly, one must also highlight that most of the published studies use CRRT intensities in the lower range of the area of best practice (1 to 2 l/hour; 14.3 to 28.5 ml/kg/hour for a 70 kg adult) [16,17,19-21,27,29,30,32,34,35,49,50], while the actual tendency in the clinical setting may be using CRRT intensities in the higher range (>30 ml/kg/hour), especially for septic patients [41,46]. In fact, a recent study by Varghese and colleagues studied the pharmacokinetics of piperacillin/tazobactam in critically ill patients with anuria/oliguria and CRRT at a median intensity of 38.5 ml/kg/hour, and reported higher drug CL (median 5.1 (interquartile range 4.2 to 6.2) l/hour) compared with other studies that used lower CRRT intensities (see Table 3 and 4) [38].

Moreover, the methodology for the calculation of CRRT intensity is not defined in most of the studies. Some of the studies report that an absolute CRRT intensity was prescribed to all patients, without being normalized to body weight. This leads to inherently variable CRRT doses, inversely proportional to the actual patient’s weight. For instance, an absolute CRRT intensity of 2 l/hour for a 100 kg patient results in a relative flow rate of 20 ml/kg/hour, whereas for a 50 kg patient the rate is 40 ml/kg/hour. When relative flow rate is prescribed, clinicians usually use body weight previous to admission or ideal body weight, and calculate the flow rate using the following formula:

$$\mathrm{Flow}\;\mathrm{rate}=\left(Q_{D}+Q_{R}\right)/\mathrm{weight}\;\left(\mathrm{kg}\right)$$

where Q_D_ is the dialysis fluid flow rate (ml/hour) and Q_R_ is the replacement fluid flow rate (ml/hour).

The rationale of this methodology is to avoid variations in the calculated flow rate over time as the patient real weight fluctuates during the ICU stay (for example, due to fluid therapy or edema) [53]. However, most of the studies do not report how body weight was considered in spite of the fact that it is essential to know which CRRT intensity was prescribed [43]. When real body weight is used, the calculated flow rate may be falsely low, as the denominator in the equation usually increases during the ICU stay. Recommendations include application of body weight previous to admission or ideal body weight [43]. However, considering the increasing prevalence of obesity in developed countries, one should discuss whether ideal body weight or body weight previous to admission should be used.

### Pharmacodynamic target for dosing recommendations

Antibiotic dosing recommendations intend to achieve a pharmacodynamic target that, for beta-lactams, is defined by the %T_>MIC_ value [54]. Classical studies report that penicillins and monobactams require at least a 50 to 60 % T_>MIC_ for maximal bactericidal activity, cephalosporins require a 60 to 70 % T_>MIC_ and carbapenems require a 40 % T_>MIC_[54]. However, most of these recommendations are based on *in vitro* studies and on animal models of bacteremia, where penetration into the site of infection is not considered. *In vivo*, higher %T_>MIC_ values in plasma may be needed for achieving the abovementioned targets in biophases other than the bloodstream, since penetration into the target site follows diffusion kinetics and depends on the physicochemistry of each particular tissue. For instance, Roberts and colleagues reported that continuous infusion of full doses of meropenem (that is, 100 % T_>MIC_ in plasma) was required for achieving 40 % T_>MIC_ for less susceptible pathogens in subcutaneous tissue [11]. Also, the attainment of a particular percentage of T_>MIC_ may be modified by the susceptibility cutoff values for the different bacteria, which vary depending on the country where the study is performed (for example, European Committee on Antimicrobial Susceptibility Testing vs Clinical and Laboratory Standards Institute breakpoints). The recommendations based upon a particular minimum inhibitory concentration in Europe may therefore not apply to the United States of America and *vice versa*.

Critical review of clinical pharmacokinetics data leads to the final consideration that there are multiple missed opportunities in the available literature. Further studies should be more focused on the study population of critically ill patients with septic shock in order to avoid variability derived from pathophysiological conditions other than septic shock. Inclusion and exclusion criteria should therefore carefully evaluate the admission diagnosis and the patient condition during the study period. Also, a population pharmacokinetics approach would be preferred to the noncompartmental approach, since the noncompartmental approach draws inaccurate conclusions because covariates that have an effect on parameter variability cannot be identified. Finally, consensus regarding clinical pharmacodynamic targets for beta-lactams would be helpful in the unification of dosing recommendations.

## Conclusions

Optimization of beta-lactam therapy in CRRT is complex and is dependent on several drug, CRRT and patient-related factors. Consideration of drug physicochemistry and protein binding, CRRT settings and disease-related pharmacokinetic alterations is essential for individualizing dose regimens with the purpose of attaining pharmacodynamic targets associated with success.

During the first day, an initial loading dose is required to achieve drug concentrations within the therapeutic range early in time, regardless of impaired organ function. This principle may also apply to the moment of CRRT commencement, where a loading dose may be required to maintain concentrations within the therapeutic range. From day 2, dosing must be adjusted to CRRT settings and residual renal function. The complexity of dosing occurs due to the great variability encountered. As such, TDM of trough levels of beta-lactams may be regarded as a promising and key tool to individualize dosing daily and to ensure optimal exposure to the antibiotic.

Current dose recommendations are based on studies with some drawbacks that limit their applicability to the current clinical scenario. Mainly, dosing recommendations in CRRT follow a one-size-fits-all fashion, despite emerging clinical data suggesting that beta-lactam CL is partially dependent on CRRT modality and intensity. Moreover, heterogeneous populations have been pooled in the studies, limiting extrapolation to critically ill patients with septic shock and CRRT. Finally, there is still some controversy on the %T_>MIC_ value that must be chosen as the pharmacodynamic target associated with success for tailoring dosing recommendations.

Further research on dose adjustment of beta-lactam antibiotics in critically ill patients with septic shock and CRRT is required in order to establish reliable and up-to-date recommendations that ensure optimal therapy and thus increase the likelihood of optimal outcomes in this population.

## Abbreviations

CL: Clearance; CRRT: Continuous renal replacement therapy; Q_D_: Dialysis fluid flow rate; Q_R_: Replacement fluid flow rate; %T_>MIC_: Percentage of dosing interval when concentration of the antibiotic is above the minimum inhibitory concentration of the pathogen; TDM: Therapeutic drug monitoring; Vd: Volume of distribution.

## Competing interests

The authors declare that they have no competing interests.
